# Effects of a cognitive–motor dual task on neuromuscular and biomechanical control during single-leg drop landing in individuals with chronic ankle instability

**DOI:** 10.3389/fpubh.2026.1912171

**Published:** 2026-07-17

**Authors:** Yanzhi He, Zilong Wang, Wenxin Sun, Huizi Cui, Bojun Zhou, Jianming Wu, Jianfu Cheng, Pengfei Wang, Ting Wang, Weipeng Liu, Yixuan Lu, Rui Li, Yuning Cheng, Chaofu Chen

**Affiliations:** 1 School of Physical Education, Jimei University, Xiamen, China; 2 Sports Science Research Institute, Jimei University, Xiamen, China; 3 Department of Sports and Leisure, Dongshin University, Naju, Republic of Korea; 4 School of Competitive Sports, Beijing Sport University, Beijing, China; 5 School of Strength and Conditioning, Shenyang Sport University, Shenyang, Liaoning, China

**Keywords:** chronic ankle instability, cognitive-motor dual task, single-leg drop landing, ground reaction force, surface electromyography, joint moments, muscleco-contraction

## Abstract

**Objective:**

To compare lower-extremity neuromuscular and biomechanical characteristics during single-leg drop landing under single-task and cognitive–motor dual-task conditions in individuals with chronic ankle instability (CAI).

**Methods:**

Twenty-three male participants with unilateral CAI completed single-leg drop landings from a 30-cm platform under single-task and auditory 2-back dual-task conditions in randomized order using a within-participant repeated-measures design. Whole-body three-dimensional kinematics, ground reaction force (GRF), and surface electromyographic (sEMG) signals from eight muscles of the affected limb were recorded synchronously. Outcomes included GRF and dynamic-stability measures, hip, knee, and ankle joint angles and internal moments, muscle activation, and co-contraction indices (CCIs). Depending on the distribution of paired differences, two-sided paired-samples *t*-tests or Wilcoxon signed-rank tests were used. Effect sizes were reported as Cohen's dz or rank-biserial correlations (rrb), respectively.

**Results:**

Compared with the single-task condition, the dual-task condition produced a higher peak vertical GRF (vGRF; *p* = 0.004, dz = 0.66) and smaller hip flexion angles at peak vGRF (*p* = 0.033, rrb = −0.507) and maximum knee flexion (*p* = 0.012, rrb = −0.587). At maximum knee flexion, the internal hip extension moment was lower (*p* < 0.001, dz = −0.80), whereas the internal ankle plantarflexion moment was higher (*p* = 0.028, dz = 0.49). The dual-task condition was also associated with lower lateral gastrocnemius root-mean-square activation (*p* = 0.002, dz = −0.75), higher peak tibialis anterior activation (*p* = 0.015, dz = 0.55), higher peak vastus lateralis activation (*p* = 0.001, dz = 0.77), and a lower peroneus longus–medial gastrocnemius CCI (*p* = 0.030, dz = −0.48). No significant between-condition differences were observed in average vertical loading rate, time to stabilization, time to peak vGRF, hip flexion at initial contact, the measured knee and ankle joint angles, or frontal-plane joint angles.

**Conclusion:**

In individuals with CAI, a concurrent working-memory task was associated with higher peak impact, reduced hip flexion at selected post-contact events, and selective changes in net internal joint moments and lower-extremity sEMG measures. These findings indicate that dual-task effects on landing control are outcome- and event-specific rather than uniform across biomechanical and neuromuscular domains.

## 1 Introduction

The ankle complex connects the lower leg to the foot, and its osseous architecture, ligamentous constraints, and multijoint motion jointly regulate ankle–foot mobility, load transmission, and postural control (1). Lateral ankle sprain is among the most common musculoskeletal injuries sustained during sport and physical activity and imposes a substantial burden through persistent symptoms and recurrence. Previous reviews have estimated that as many as 70% of individuals may develop CAI after an acute lateral ankle sprain, whereas prospective evidence indicates a prevalence of approximately 40% 1 year after a first-time lateral ankle sprain (2). CAI is commonly characterized by recurrent ankle sprains, episodes of “giving way” or perceived instability, pain, swelling, restricted activity, weakness, or self-reported functional impairment persisting for more than 12 months after the index injury (3). CAI encompasses mechanical impairments, such as residual pathological joint laxity; sensory–perceptual deficits, such as perceived instability and recurrent giving-way episodes; and sensorimotor alterations involving proprioception and neuromuscular control. These interrelated deficits may prompt adaptive motor-control strategies during dynamic tasks such as landing and jumping (3, 4).

Early conceptual models proposed that CAI may arise from mechanical instability, functional instability, or their coexistence, with functional instability being attributed primarily to deficits in proprioception and neuromuscular control (5). Subsequent models broadened this framework by emphasizing interactions among the primary tissue injury, pathological mechanical impairments, sensory–perceptual deficits, motor–behavioral deficits, and personal and environmental factors. This broader perspective suggests that persistent instability in a substantial proportion of individuals cannot be explained solely by localized ligamentous laxity or an isolated peripheral joint abnormality (2, 3, 6). Crucially, mounting evidence indicates that the consequences of unilateral lateral ankle sprains extend well beyond the injured limb, driving supraspinal and central sensorimotor adaptations. These central neuroplastic changes often manifest as bilateral impairments in postural control and altered global sensorimotor organization, reinforcing the concept that CAI is a systemic motor control issue rather than a strictly local defect (7, 8). Because CAI involves such widespread central reorganization, dynamic stabilization becomes less automatic and more reliant on higher-level cognitive processing. The International Ankle Consortium similarly recommends that CAI research samples include a clearly documented history of a substantial ankle sprain, recurrent instability or giving-way episodes, and self-reported functional limitations, thereby reducing heterogeneity caused by combining distinct clinical states (9). Beyond peripheral sensorimotor deficits, individuals with CAI may also exhibit differences in attention, visual memory, and other neurocognitive domains (10). A systematic review and meta-analysis further indicated that dual-task training may improve static and dynamic postural stability in this population (11). Accordingly, investigations of motor control in CAI should extend beyond static balance and isolated ankle assessments to dynamic functional tasks that simultaneously challenge rapid load attenuation, single-leg support, postural recovery, and cognitive–motor integration (3, 11).

Single-leg drop landing is well-suited to evaluating dynamic stability in individuals with CAI because it requires rapid impact attenuation, joint stabilization, and postural recovery during single-leg support while exposing the ankle–foot complex and lower limb to impact loads of approximately two to five times body weight (BW) (12–14). Systematic reviews indicate that individuals with CAI may display impaired dynamic postural stability, higher peak GRF values and loading rates, and altered lower-extremity biomechanics during single-leg landing, suggesting that high-impact tasks may reveal motor-control differences more readily than static standing or low-intensity walking (13, 15). GRFs represent the external exchange of load between the body and the ground, and individuals with functional ankle instability may exhibit alterations in both force magnitude and the timing of peak force during jump landing (16). Joint kinematics describe joint position and motion, whereas joint kinetics reflect the distribution of internal loading. In a sample of 30 elite athletes, Lin et al. (17) reported greater hip flexion angles at IC and at the lowest landing position in the CAI group than in healthy controls. The same study identified an ankle inversion angular velocity of approximately −220°/s during the early post-contact phase, together with lower PL and TA activation, suggesting that altered joint motion may coexist with insufficient neuromuscular control around the ankle (17). Similarly, Delahunt et al. (12) observed concurrent differences in three-dimensional kinematics, kinetics, and ankle-muscle activity during single-leg drop landing in 24 participants with functional ankle instability and 24 healthy controls, indicating that CAI-related alterations are not confined to a single joint angle or muscle. During the landing-descent, or eccentric-deceleration, phase after IC, the hip, knee, and ankle joints and their associated musculature must collectively absorb downward momentum and stabilize single-leg support; GRFs, joint angles, joint moments, and muscle activity are therefore interdependent (18, 19). Integrating these measures may clarify whether individuals with CAI adopt distinct internal load-distribution and neuromuscular strategies despite similar observable landing postures, thereby providing a biomechanical basis for examining the influence of cognitive load on landing control (12, 18).

Postural control is not an entirely automatic reflexive process; rather, it is influenced by the allocation of attentional resources and by cognitive load, particularly when task difficulty increases or sensory information is constrained (20). Within a postural–cognitive dual-task framework, concurrently performed postural and cognitive tasks compete for finite attentional resources and may consequently alter postural stability or movement-execution strategies (20, 21). Individuals with CAI may exhibit not only alterations in proprioception, postural control, and gait mechanics, but also differences in attention, visual memory, and cognitive processing (10, 22). A systematic review by Choi et al. (22) that included nine dual-task studies in CAI found no consistent effect of cognitive loading on postural control, indicating that the available evidence is sensitive to task selection and outcome definition. More recent work has incorporated cognitive tasks into high-impact single-leg landing. Wang et al. (23) found that a cognitive task altered selected joint angles, peak vGRF, and loading rate in individuals with functional ankle instability, suggesting that cognitive–motor interference may affect landing impact and motor-control strategies. That study, however, focused primarily on joint kinematics and GRFs, leaving internal joint loading and neuromuscular regulation comparatively underexplored. The 2-back task is a canonical n-back working-memory paradigm that requires continuous updating, maintenance, and comparison of sequential information, thereby imposing sustained attentional and working-memory demands (24). In the present study, an auditory 2-back task was used to impose working-memory load without requiring participants to continuously monitor an additional visual display, thereby minimizing direct interference with visual surveillance of the landing environment. Combining an auditory 2-back task with single-leg drop landing, while concurrently examining impact, joint posture, internal joint moments, muscle activation, and co-contraction, may provide a more comprehensive account of multilevel cognitive–motor regulation in individuals with CAI (12, 20, 23).

Taken together, individuals with CAI may demonstrate sensorimotor, biomechanical, and neuromuscular-control alterations during postural, gait, and single-leg landing tasks, and cognitive loading may further modify their motor-control strategies (3, 13, 22). Nevertheless, existing dual-task research in CAI has focused predominantly on balance, gait, or global postural stability, and the integrated behavior of GRFs, lower-extremity kinematics and kinetics, and muscular coordination during high-impact single-leg drop landing remains insufficiently understood. The present study therefore compared GRFs, dynamic stability, hip, knee, and ankle joint angles and internal moments, lower-extremity muscle activation, and co-contraction during single-leg drop landing under single-task and auditory 2-back dual-task conditions in individuals with CAI. It was hypothesized that the two conditions would differ in (1) GRF and dynamic-stability measures; (2) hip, knee, or ankle kinematics and kinetics; and (3) activation and co-contraction of muscles surrounding the ankle and acting across the knee.

## 2 Participants and methods

### 2.1 Participants

Twenty-three male university students with unilateral CAI completed the entire experimental protocol and were included in the statistical analyses. All participants regularly engaged in recreational sport; most played basketball at an amateur level, and some participated in badminton. A sensitivity analysis for a two-sided paired-samples *t*-test was conducted in G^*^Power 3.1.9.2 (25, 26). With *n* = 23, α = 0.05, and statistical power of 0.80, the minimum detectable effect size was Cohen's dz = 0.61 (27). The study was conducted in accordance with the ethical principles of the 2024 revision of the Declaration of Helsinki (28) and was formally approved by the Institutional Ethics Committee of Jimei University (approval no. 20260526001). Participant characteristics are presented in Table 1.

**Table 1:** Participant characteristics (*n* = 23).

Characteristic	Mean ±standard deviation
Age (years)	20.0 ± 0.9
Height (cm)	178.6 ± 6.9
Body mass (kg)	74.8 ± 9.4
Body mass index (BMI) (kg/m^2^)	23.4 ± 1.7
CAIT score (points)	19.1 ± 2.8

Values are presented as mean ± standard deviation.

To enhance the consistency of sample definition and the comparability of findings across studies, participants were screened in accordance with the International Ankle Consortium guidelines and contemporary CAI research criteria (3, 9, 22, 29). Eligibility was verified from screening records completed before testing. Inclusion criteria were as follows: (1) at least one substantial lateral ankle sprain sustained ≥12 months before testing, accompanied by inflammatory symptoms such as pain and swelling and resulting in interruption of physical activity for at least 1 day; (2) a history of recurrent ankle sprains and episodes of ankle “giving way” or persistent perceived instability during basketball-specific movements or other sporting activities; and (3) a Cumberland Ankle Instability Tool (CAIT) score ≤ 24. Exclusion criteria were as follows: (1) bilateral CAI; (2) a history of lower-extremity fracture or orthopedic surgery, or any acute bone, muscle, or ligament injury within the 6 months preceding testing; (3) concurrent participation in another physical rehabilitation intervention that could influence the study outcomes; (4) any known vestibular, visual, or neurological disorder affecting postural balance or dynamic stability; (5) any psychological, psychiatric, or cognitive condition that impeded understanding or completion of the 2-back working-memory task; (6) a Montreal Cognitive Assessment (MoCA) score <26, indicating possible cognitive impairment (30); and (7) inability to complete the single-leg drop-landing task as instructed, or missing or inadequate-quality sEMG, three-dimensional kinematic, or GRF data.

### 2.2 Instrumentation

Three-dimensional kinematic data were collected at 200 Hz using a 14-camera motion-analysis system comprising four Arqus cameras and 10 Miqus cameras (Qualisys AB, Gothenburg, Sweden). Forty-one retroreflective markers were positioned in strict accordance with the official Qualisys Motion Marker Set—Functional Assessment protocol. GRF data were synchronously acquired at 1,000 Hz using two custom embedded three-dimensional piezoelectric force plates (9287CA, Kistler Group, Winterthur, Switzerland). sEMG signals from the tested lower limb were recorded at 2,000 Hz using a wireless Cometa system equipped with MiniWave sensors and a Wave Plus receiver (Cometa S.r.l., Bareggio, Milan, Italy). The eight recorded muscles were the rectus femoris (RF), vastus lateralis (VL), biceps femoris (BF), semitendinosus (ST), tibialis anterior (TA), peroneus longus (PL), medial gastrocnemius (MG), and lateral gastrocnemius (LG) (12, 31). Kinematic, kinetic, and sEMG signals were hardware-synchronized and integrated in Qualisys Track Manager to ensure complete temporal alignment. Skin preparation and electrode placement followed the Surface Electromyography for the Non-Invasive Assessment of Muscles (SENIAM) recommendations (32). Hair was removed when necessary, the skin was lightly abraded and cleaned with alcohol, and, once dry, bipolar wireless sEMG sensors were affixed parallel to the muscle-fiber direction to reduce skin impedance and crosstalk from adjacent muscles (32, 33).

### 2.3 Research design and procedure

Before experimental testing and data collection, all participants were fully informed of the study purpose, procedures, potential risks, and benefits and provided written informed consent. All biomechanical and physiological data were rigorously anonymized and used exclusively for academic research. Screening and experimental procedures were administered by personnel who had received standardized training to minimize variability in eligibility assessment, marker placement, and task execution (9, 32). Participants completed a standardized warm-up and were familiarized with the single-leg drop-landing and 2-back working-memory tasks to reduce learning effects during formal testing (23). The affected limb was tested in all participants to evaluate its neuromuscular and biomechanical control under the cognitive–motor dual-task condition (3, 9). Skin preparation and electrode placement for sEMG were performed first. Forty-one retroreflective markers were then attached in accordance with the Qualisys Functional Assessment whole-body model, and a static standing calibration trial was collected to construct the whole-body segment model. Before the dynamic tasks, maximum voluntary isometric contractions (MVICs) of the eight target muscles were recorded for normalization of sEMG amplitude (34). For each target muscle group, participants completed three 5-s maximal isometric contractions against fixed manual resistance applied by the investigator, with 60 s of rest between trials to minimize neuromuscular fatigue. Standardized testing positions were used: seated resisted knee extension for the RF and VL; prone resisted knee flexion for the BF and ST; seated resisted ankle dorsiflexion for the TA; seated resisted ankle eversion for the PL; and prone resisted ankle plantarflexion for MG and LG. Three-dimensional kinematics, GRFs, and sEMG were recorded synchronously during formal testing. Condition order was determined using a computer-generated random sequence: 12 participants completed the single-task condition first, and 11 completed the dual-task condition first. A 3-min rest interval separated the two conditions. Three technically valid trials with complete signals were collected in each condition, and the mean of the three trials was used for statistical analysis.

Under the single-task condition, participants stood with both feet on a 30-cm-high platform and placed their hands on their hips to minimize the influence of arm swing. They stepped naturally from the platform with the tested limb and descended under gravity; active upward jumping and deliberate downward acceleration were prohibited. Participants landed with the entire affected foot centered on the force plate, completed the landing using their habitual strategy, and stabilized as rapidly as possible while maintaining quiet single-leg stance for at least 5 s (12, 13, 35). A trial was repeated if the participant hopped after landing, moved the supporting foot, touched the ground with the contralateral limb, removed either hand from the hips, failed to place the tested foot fully on the force plate, or failed to maintain the prescribed posture (12).

The dual-task condition combined single-leg drop landing with a continuous auditory 2-back working-memory task. The task comprised 25 pseudorandom auditory letter stimuli selected from A to J. Each stimulus lasted 400 ms, the interstimulus interval was 3,000 ms, and 20% of the stimuli were targets. While standing on the 30-cm platform, participants verbally indicated whether the current letter matched the letter presented two positions earlier, thereby continuously updating and maintaining short-term information (24). An asynchronous paradigm was used: during the ongoing 2-back sequence, an 800-Hz auditory cue lasting 500 ms was presented at a random time. Participants initiated the single-leg drop landing immediately after the cue while continuing the 2-back task (20, 23). The dual-task trial was repeated if 2-back accuracy was <80%, verbal response time exceeded 2,000 ms, or the landing failed to satisfy the criteria for a valid trial (11, 22).

### 2.4 Data processing and analysis

All hardware-synchronized three-dimensional kinematic, GRF, and sEMG data were processed using Visual3D 2021a (C-Motion, Inc., Germantown, MD, USA) and custom scripts. A whole-body rigid-segment model was constructed from the 41-marker dataset and the static calibration trial, and whole-body center-of-mass (COM) position was calculated from this model. A dual-channel filtering strategy was adopted to accommodate the inherent differences in frequency content between kinematic and kinetic signals during high-dynamic-impact movements. For inverse-dynamics calculations, marker trajectories and GRFs were smoothed using identical fourth-order, zero-lag Butterworth low-pass filters with a 15-Hz cutoff frequency. This procedure reduced temporal and frequency-content mismatches between the two signal classes and mitigated artificial impact peaks in joint-moment curves (36, 37). By contrast, when discrete kinetic variables—including peak vGRF, time to peak vGRF, and LR—were extracted, GRF data were independently processed with a fourth-order, zero-lag Butterworth low-pass filter at 50 Hz to preserve, as far as possible, high-frequency components of the impact transient (37, 38).

Hip, knee, and ankle joint angles were calculated using local joint coordinate systems, with static standing defined as 0°. The X-axis represented sagittal-plane motion: positive values denoted hip flexion, knee flexion, and ankle dorsiflexion, whereas negative values denoted the opposite directions. The Y-axis represented frontal-plane motion: positive values denoted hip adduction, knee varus, and ankle inversion, whereas negative values denoted hip abduction, knee valgus, and ankle eversion. Internal hip, knee, and ankle joint moments were calculated using inverse dynamics and normalized to body mass, with units of N·m/kg (12, 18). Positive X-axis moments represented internal hip extension, knee extension, and ankle plantarflexion moments, respectively; positive Y-axis moments represented internal hip adduction, knee varus, and ankle inversion moments. Negative values denoted moments in the opposite directions.

IC was defined as the instant at which the vGRF first exceeded 10 N (38). The landing-descent phase was strictly defined as the interval from IC to the minimum vertical position of the whole-body COM, with the COM minimum corresponding to the instant at which vertical velocity reached zero (18). Maximum knee flexion was defined as the instant at which the knee X-axis angle reached its maximum between IC and the COM minimum. Peak vGRF was defined as the maximum vGRF during the landing-descent phase and was expressed in multiples of BW. Time to peak vGRF was defined as the interval from IC to peak vGRF. Peak vGRF and time to peak vGRF were identified from the GRF channel low-pass filtered at 50 Hz; joint angles and internal moments corresponding to peak vGRF were extracted at the same event time from the kinematic and inverse-dynamics channels processed at 15 Hz, respectively. Kinematic and kinetic variables were also extracted at IC and at the maximum-knee-flexion event defined above. LR was calculated by dividing peak vGRF by the interval from IC to peak vGRF and was expressed in BW/s.

TTS was calculated from GRF data low-pass filtered at 50 Hz using a fixed-force-threshold method (39). Beginning at IC, the earliest common onset at which the normalized anterior–posterior and medial–lateral GRFs remained within 0 ± 0.025 BW and the vGRF remained within 1.00 ± 0.05 BW was identified. All three components were required to satisfy their respective thresholds simultaneously from the same instant and to remain within those thresholds continuously for at least 3 s. TTS was defined as the interval from IC to this common onset of stabilization. Each trial was recorded for at least 5 s after contact to ensure that the recording extended sufficiently beyond the identified stabilization time and to verify that all three GRF components remained continuously within their prescribed thresholds throughout the required 3-s interval.

Raw sEMG signals were first band-pass filtered at 20–450 Hz and notch filtered at 50 Hz to attenuate movement artifact, high-frequency noise, and mains interference. The mean was then removed to correct baseline drift, and the signals were full-wave rectified (33). Rectified signals were subsequently processed with a fourth-order, zero-lag Butterworth low-pass filter at 20 Hz to obtain the sEMG linear envelope (33, 34). The sEMG analysis window corresponded to the landing-descent phase, from IC to the minimum vertical position of the whole-body COM.

RMS amplitude was calculated over the landing-descent phase from the band-pass-filtered, notch-filtered, and demeaned sEMG signals. For RMS normalization, the RMS value was calculated for each of the three MVIC trials, and the largest value was taken as the 100% MVIC reference for the corresponding muscle. The task RMS value for each muscle was divided by this reference and expressed as %MVIC.

The absolute peak was extracted from the smoothed linear envelope of each of the three MVIC trials, and the largest of these peaks was used as the 100% MVIC reference for the corresponding muscle. sEMG linear envelopes from the dynamic tasks were amplitude-normalized to the corresponding MVIC reference. Before discrete variables such as peak activation and the CCI were extracted, the normalized sEMG linear envelopes were time-normalized to 101 data points spanning 0–100% of the landing-descent phase. Peak activation was defined as the maximum amplitude of the time-normalized linear envelope during the landing-descent phase and was expressed as %MVIC.

RMS amplitude and peak activation were calculated for the RF, VL, BF, ST, TA, PL, MG, and LG. RMS amplitude represented overall sEMG amplitude across the entire landing-descent phase, whereas peak activation represented the maximum amplitude of the normalized linear envelope within that phase.

A CCI was used to quantify co-contraction of functionally related muscle pairs during the landing-descent phase. Knee-related muscle pairs comprised RF–BF, RF–ST, VL–BF, and VL–ST. Ankle-related pairs comprised TA–MG, TA–LG, TA–PL, and PL–MG (12, 31). The CCI was calculated point by point from the MVIC-normalized sEMG linear envelopes and was then averaged across the entire landing-descent phase:


$$\begin{array}{l} \text{CCI }=\text{ Σ }\left[2\;\times \;\text{min}\left({\text{EMG1}}_{\text{i}},{\text{EMG2}}_{\text{i}}\right)/\left({\text{EMG1}}_{\text{i}}\;+\;{\text{EMG2}}_{\text{i}}\right)\right]/\text{N} \end{array}$$

where EMG1_i_ and EMG2_i_ denote the amplitudes of the normalized linear envelopes of the two muscles at time point i, and N denotes the number of time-normalized data points. A higher CCI indicates a greater relative overlap in activation between the two muscles.

The outcome measures were categorized as follows. GRF and dynamic-stability outcomes: (1) LR; (2) TTS; (3) peak vGRF; and (4) time to peak vGRF.

Kinematic outcomes: (1) hip joint angles; (2) knee joint angles; and (3) ankle joint angles in the X and Y axes at IC, peak vGRF, and maximum knee flexion (18 variables).

Kinetic outcomes: (1) internal hip joint moments; (2) internal knee joint moments; and (3) internal ankle joint moments in the X and Y axes at IC, peak vGRF, and maximum knee flexion (18 variables).

sEMG outcomes: (1) RMS amplitude for the eight recorded muscles; (2) peak activation for the eight recorded muscles; and (3) the eight CCIs described above.

### 2.5 Statistical analysis

All statistical analyses were performed in Python 3.10. NumPy 1.26.4 and pandas 2.2.3 were used for data organization and numerical computation; SciPy 1.15.3 was used for the Shapiro–Wilk test, two-sided paired-samples *t*-tests, and two-sided Wilcoxon signed-rank tests; and statsmodels 0.14.4 was used to generate quantile–quantile (Q–Q) plots. Effect sizes and confidence intervals were calculated using custom Python functions. Analyses compared paired observations obtained from the same participants with CAI under the single-task and auditory 2-back dual-task conditions. Paired differences were defined as dual-task minus single-task values (27). Normality of the paired differences was assessed using the Shapiro–Wilk test in conjunction with visual inspection of Q–Q plots (40). Outcomes for which paired differences approximated a normal distribution were compared using two-sided paired-samples *t*-tests; outcomes that clearly violated the normality assumption were compared using two-sided Wilcoxon signed-rank tests. For paired-samples *t*-tests, the mean and standard deviation for each condition, the mean paired difference, 95% confidence interval (CI), t statistic, degrees of freedom, exact *p*-value, and Cohen's dz were reported. Cohen's dz was defined as the mean paired difference divided by the standard deviation of the paired differences (27). For Wilcoxon signed-rank tests, the median and interquartile range (IQR), test statistic, exact *p*-value, and r_rb were reported. Statistical significance was set at α = 0.05.

## 3 Results

### 3.1 RMS muscle activation

**Figure 1:**
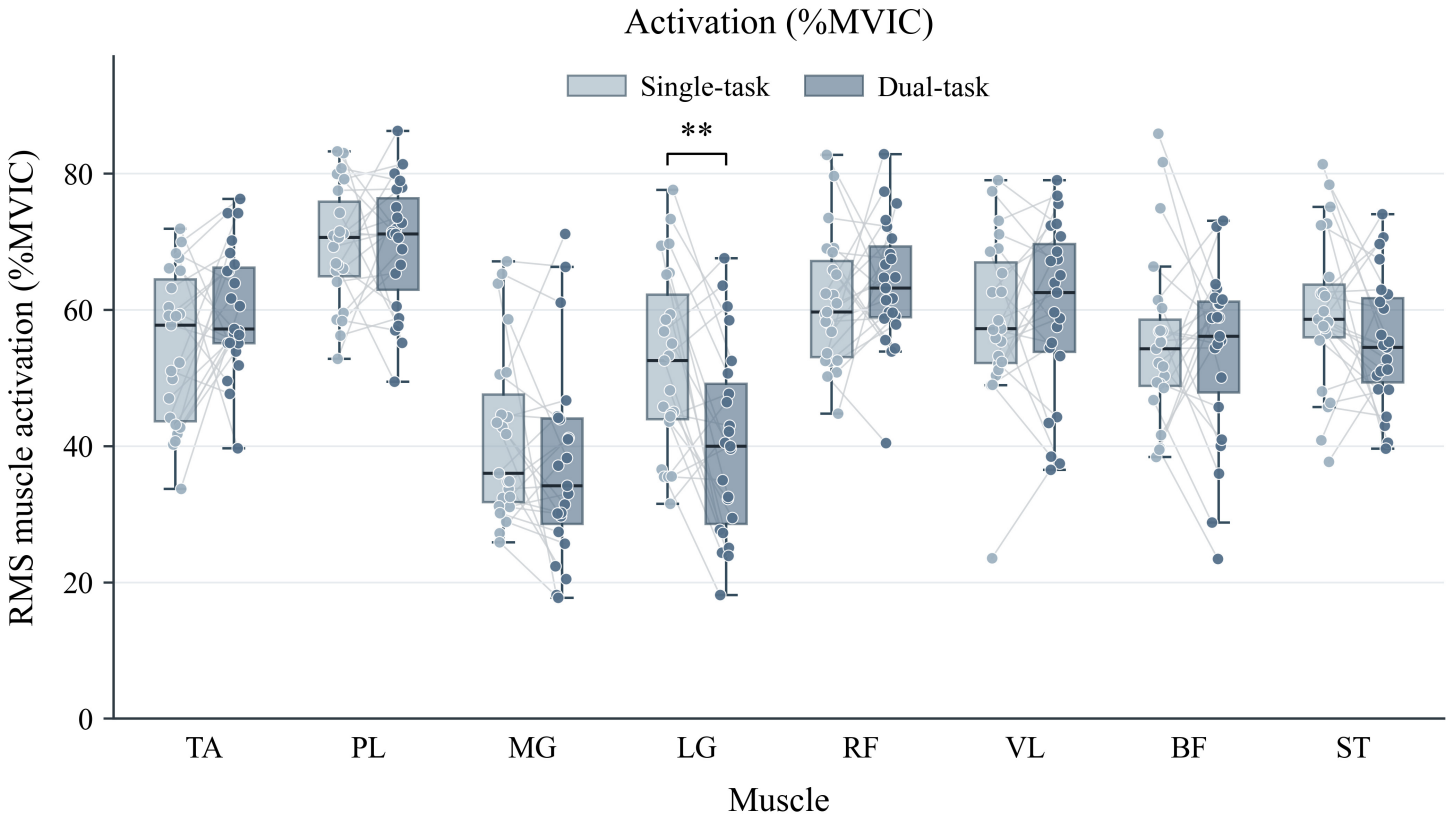
RMS muscle activation during the landing-descent phase under the single-task and auditory 2-back dual-task conditions. **p < 0.01.

Compared with the single-task condition, RMS activation of LG was lower under the auditory 2-back dual-task condition (*p* = 0.002, Cohen's dz = −0.75). No statistically significant differences were detected for the remaining RMS outcomes (Figure 1).

### 3.2 Peak muscle activation

**Figure 2:**
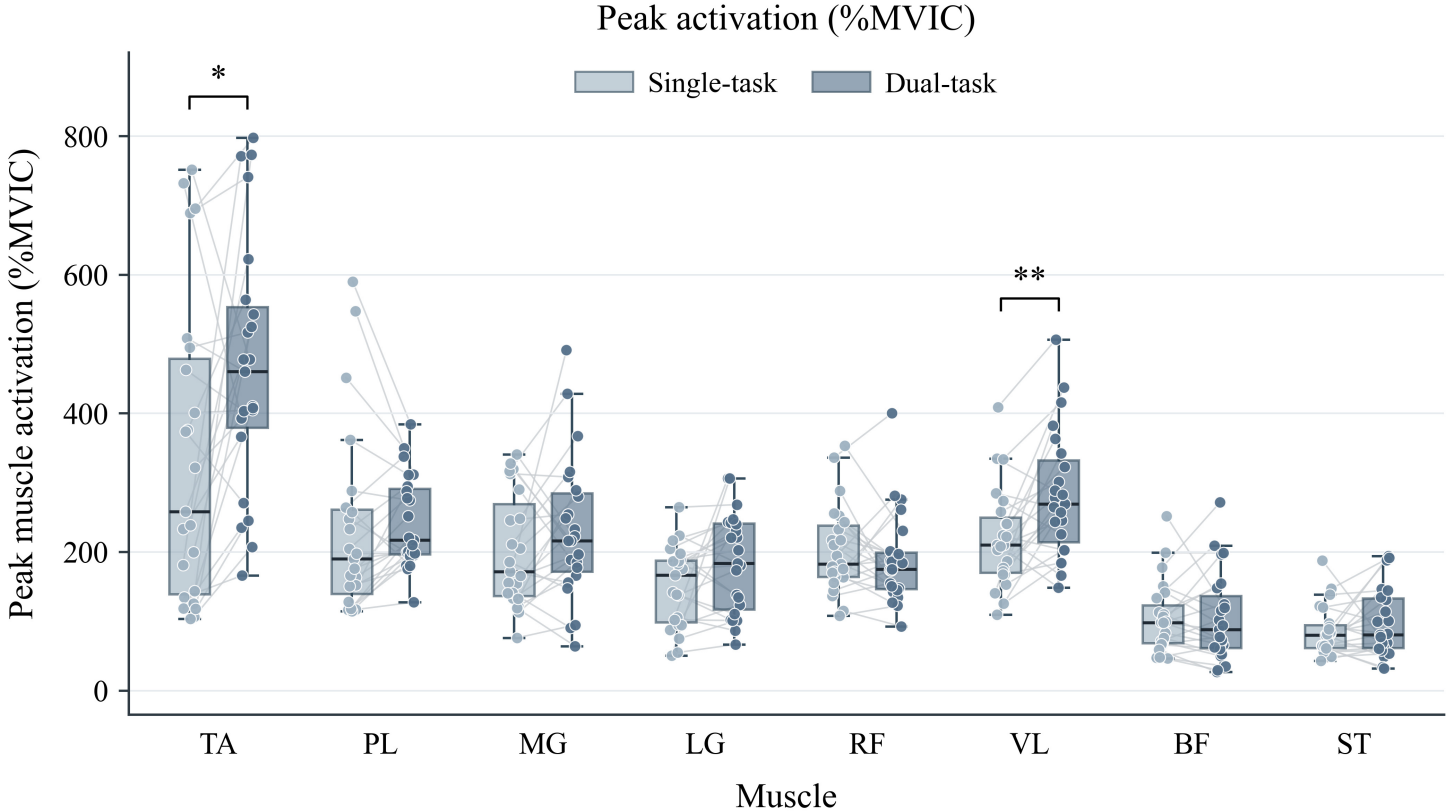
Peak muscle activation during the landing-descent phase under the single-task and auditory 2-back dual-task conditions. *p < 0.05; **p < 0.01.

Compared with the single-task condition, peak activation of VL was higher under the auditory 2-back dual-task condition (*p* = 0.001, Cohen's dz = 0.77), as was peak activation of TA (*p* = 0.015, Cohen's dz = 0.55). No statistically significant differences were detected for the remaining peak-activation outcomes (Figure 2).

### 3.3 CCI

**Figure 3:**
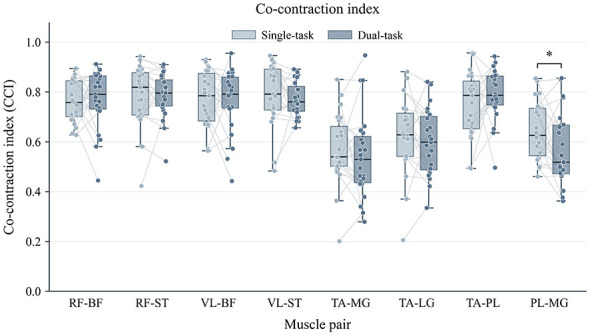
CCIs during the landing-descent phase under the single-task and auditory 2-back dual-task conditions. *p < 0.05.

Compared with the single-task condition, the PL–MG CCI was lower under the auditory 2-back dual-task condition (*p* = 0.030, Cohen's dz = −0.48). No statistically significant differences were detected in the CCIs of the remaining knee- or ankle-related muscle pairs (Figure 3).

### 3.4 GRF and dynamic stability

**Figure 4:**
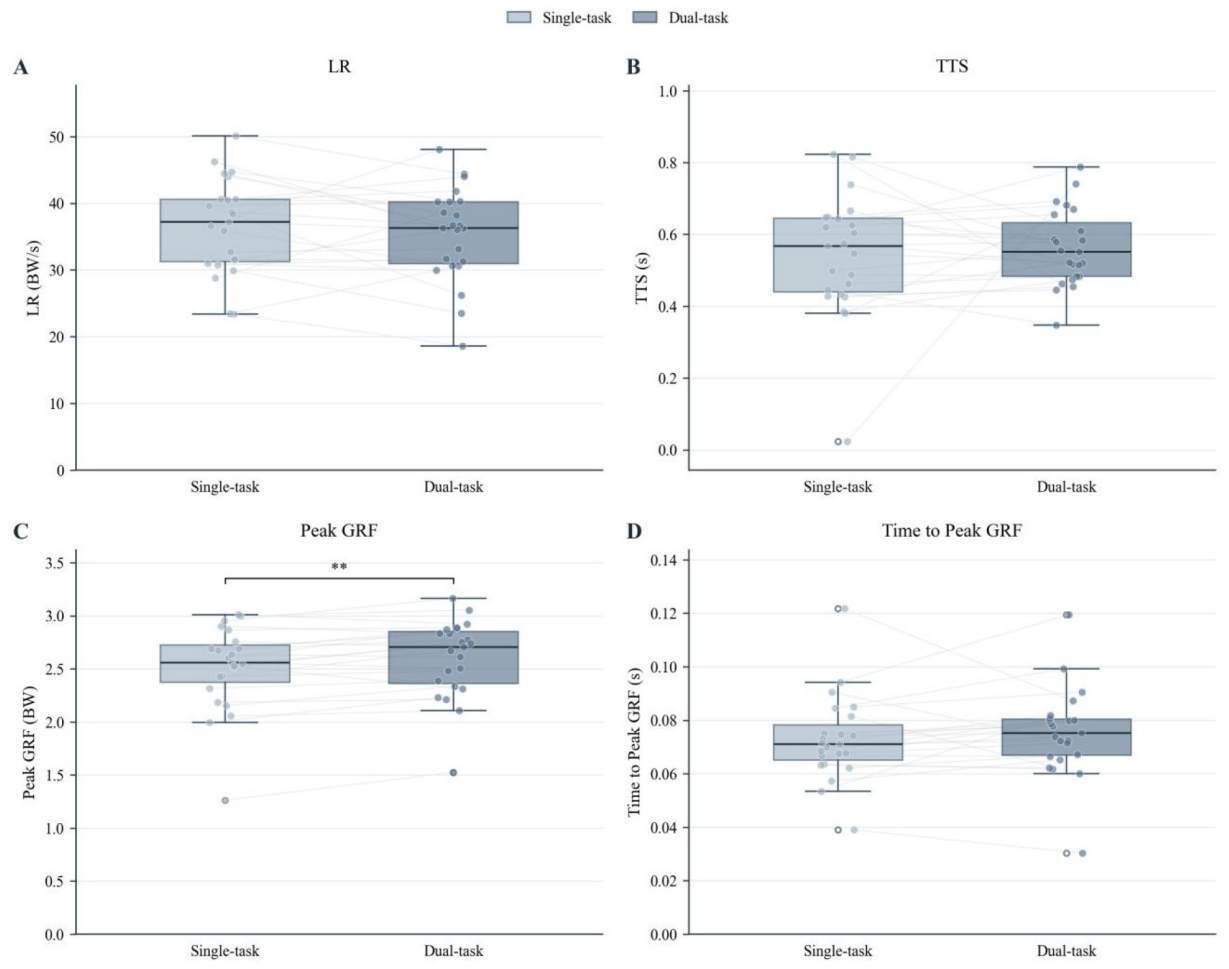
GRF and dynamic-stability measures during single-leg landing under the single-task and auditory 2-back dual-task conditions. **(A)** Average vertical loading rate(LR, BW/s); **(B)** time to stabilization (TTS, s); **(C)** peak vertical ground reaction force (peak vGRF, BW); and **(D)** time to peak vGRF (s). **p < 0.01.

Peak vGRF was higher under the auditory 2-back dual-task condition (2.60 ± 0.37 BW) than under the single-task condition (2.51 ± 0.40 BW; *p* = 0.004, Cohen's dz = 0.66). No statistically significant between-condition differences were detected in LR, TTS, or time to peak vGRF (Figure 4).

### 3.5 Lower-extremity joint angles

**Figure 5:**
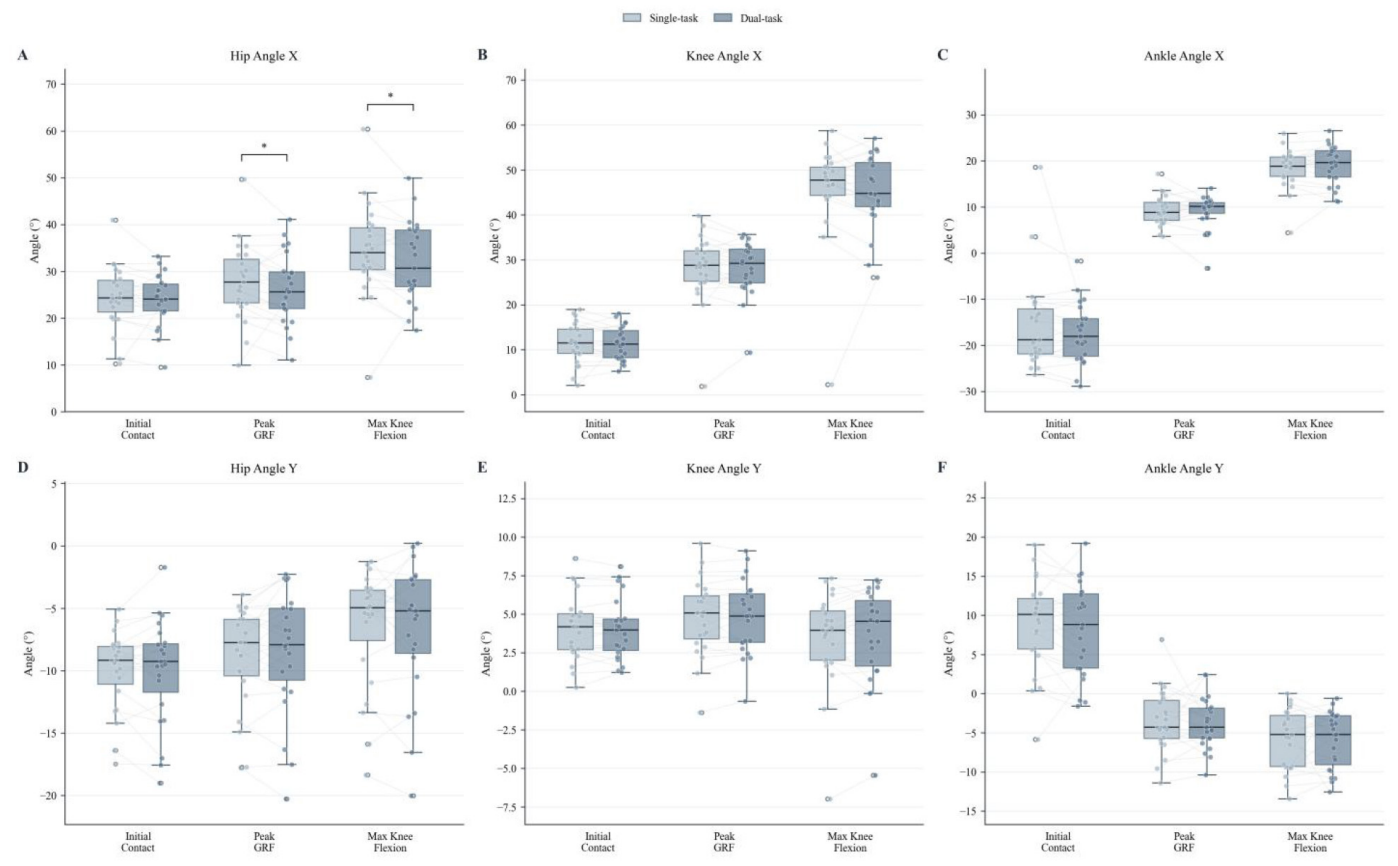
Hip, knee, and ankle joint angles at IC, peak vGRF, and maximum knee flexion under the single-task and auditory 2-back dual-task conditions. **(A–C)** show the X-axis angles of the hip, knee, and ankle, respectively, and **(D–F)** show the corresponding Y-axis angles. *p < 0.05.

Under the auditory 2-back dual-task condition, the hip flexion angle was smaller at peak vGRF (*p* = 0.033, r_rb = −0.507) and at maximum knee flexion (*p* = 0.012, r_rb = −0.587) than under the single-task condition. No statistically significant between-condition differences were detected in hip flexion at IC, knee or ankle angles at any of the three selected events, or frontal-plane joint angles (Figure 5).

### 3.6 Lower-extremity internal joint moments

**Figure 6:**
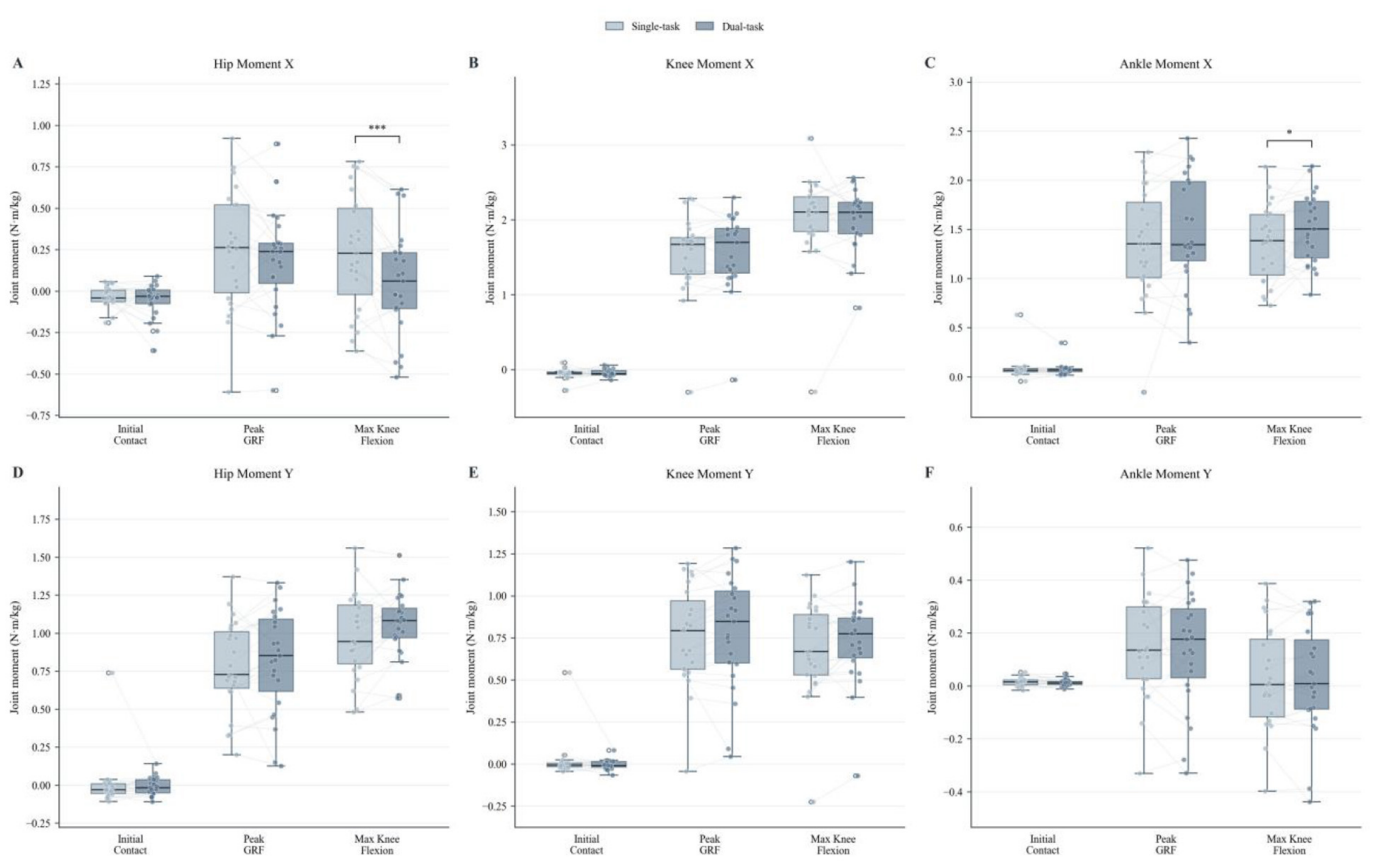
Internal hip, knee, and ankle joint moments at IC, peak vGRF, and maximum knee flexion under the single-task and auditory 2-back dual-task conditions. **(A–C)** show the X-axis moments of the hip, knee, and ankle, respectively, and **(D–F)** show the corresponding Y-axis moments. *p < 0.05; ***p < 0.001.

At maximum knee flexion, the internal hip extension moment was lower under the auditory 2-back dual-task condition than under the single-task condition (*p* < 0.001, Cohen's dz = −0.80), whereas the internal ankle plantarflexion moment was higher (*p* = 0.028, Cohen's dz = 0.49). No statistically significant differences were detected in the remaining internal joint moments at any of the three discrete events (Figure 6).

## 4 Discussion

This study examined whether landing impact, dynamic stability, lower-extremity kinematics and kinetics, and neuromuscular outcomes differed between single-task and auditory 2-back dual-task single-leg drop landings in participants with CAI. Relative to the single-task condition, peak vGRF was higher under the dual-task condition, and hip flexion angles were smaller at peak vGRF and maximum knee flexion. At maximum knee flexion, the internal hip extension moment was lower, whereas the internal ankle plantarflexion moment was higher. No statistically significant differences were detected in LR, time to peak vGRF, TTS, hip flexion at IC, the measured knee and ankle angles, frontal-plane joint angles, or the remaining internal joint moments. Under the dual-task condition, LG RMS activation and PL–MG co-contraction were lower, whereas peak TA and VL activation were higher; most other muscle-activation and CCI outcomes did not differ significantly. Thus, all three hypotheses were partially supported. Overall, the dual-task differences were outcome- and event-specific, being expressed in peak impact, selected sagittal-plane hip postures, net internal joint moments, and selected muscle-activation and co-contraction measures rather than as uniform changes across all biomechanical and neuromuscular outcomes.

The first hypothesis was supported primarily by the higher peak vGRF, whereas LR, time to peak vGRF, and TTS showed no statistically significant differences. This pattern indicates a condition-related difference in early impact magnitude without detectable differences in the selected force-development and stability measures. The effect size for peak vGRF was Cohen's dz = 0.66, representing a moderate within-participant effect. Caulfield and Garrett (16) reported that individuals with functional ankle instability may exhibit changes in GRF magnitude and peak-force timing during jump landing, interpreting these findings as evidence of impaired early landing regulation. In contrast to such between-group comparisons with healthy controls, the present within-participant design characterized differences that emerged when a concurrent cognitive task was added to the established landing behavior of participants with CAI. The higher peak vGRF therefore demonstrates that maximum vertical loading differed between conditions but does not identify the underlying control mechanism. A postural–cognitive framework offers one possible explanation because cognitive and motor tasks may compete for finite information-processing resources, although attentional allocation was not measured directly in the present study (20). The absence of significant differences in LR and time to peak vGRF indicates that the higher peak was not accompanied by detectable changes in these related temporal measures. Likewise, the absence of a TTS difference suggests that the higher early impact did not coincide with prolonged global postural recovery. This interpretation is consistent with previous reports that cognitive loading produces broadly similar changes in postural stability in individuals with CAI and healthy controls (21, 41). Peak vGRF and TTS characterize distinct temporal components of landing: the former reflects the transient impact shortly after contact, whereas the latter reflects recovery over a longer timescale (13, 16). The present findings therefore suggest that impact and stability outcomes may respond differently when a concurrent working-memory task is imposed.

The second hypothesis was partially supported at the kinematic level. Relative to the single-task condition, hip flexion was smaller at peak vGRF and maximum knee flexion under the dual-task condition, whereas no statistically significant differences were detected in hip flexion at IC, the measured knee and ankle angles, or frontal-plane joint angles. These findings indicate that kinematic differences under working-memory load emerged at two discrete post-contact events and were confined to sagittal-plane hip posture rather than constituting a generalized alteration in hip, knee, and ankle kinematics. The smaller hip flexion angles at both events reflect a relatively more extended hip configuration under the dual-task condition. Active hip and knee flexion during landing has been associated with attenuation of GRFs, although a joint angle measured at a single discrete event cannot independently determine impact magnitude (42). Consequently, although smaller hip flexion and higher peak vGRF occurred concurrently in the present study, the former cannot be inferred to have directly caused the latter. The findings partially accord with those of Wang et al. (23), who reported that a cognitive task altered selected joint angles during single-leg drop landing in individuals with functional ankle instability, suggesting that cognitive-load effects on kinematics may be event- and outcome-specific. Nevertheless, the present study identified hip-flexion differences at only two post-contact events and did not observe widespread three-dimensional changes across the hip, knee, and ankle, a pattern that is not entirely consistent with the broader landing-kinematic differences previously reported in functional ankle instability or CAI (12, 17). This discrepancy may first reflect the comparative framework: earlier studies predominantly compared CAI groups with healthy controls and therefore captured persistent between-group movement-pattern differences, whereas the present within-participant design evaluated the conditional effect of adding cognitive load to an established CAI landing pattern. The mechanical demands of the task may also be relevant. Compared with a vertical step-off landing from 30 cm, forward jump landing entails greater horizontal momentum and directional-control demands and may therefore elicit more pronounced multijoint and multiplanar kinematic differences (17, 18). Furthermore, only three discrete events—IC, peak vGRF, and maximum knee flexion—were analyzed; event-based analysis may not detect transient waveform differences occurring elsewhere during landing (43). The present findings should therefore be interpreted as smaller hip flexion at two specific post-contact events under the dual-task condition, rather than as evidence of a generalized alteration in lower-extremity landing kinematics. The second hypothesis was also partially supported at the kinetic level. At maximum knee flexion, the internal hip extension moment was lower and the internal ankle plantarflexion moment was higher, indicating opposite condition-related differences in these two net joint moments at that discrete event. During single-leg landing, the hip extensors, knee extensors, and ankle plantarflexors collectively resist lower-extremity flexion and decelerate the downward motion of the COM; their kinetic contributions are therefore interdependent (18, 19). Using an electromyography-driven neuromusculoskeletal analysis, Maniar et al. (18) identified the gluteus maximus, quadriceps, soleus, and gastrocnemius as major contributors to resisting lower-extremity flexion and downward momentum during single-leg landing. The opposing hip- and ankle-moment differences observed here should be interpreted as condition-related differences in net internal moments at maximum knee flexion, not as direct evidence of mechanical-energy transfer or redistribution. A net internal joint moment reflects the combined actions of muscles, passive tissues, and external loading; it cannot be attributed to a single muscle or equated with mechanical work at the joint (18). Because joint power and work were not calculated, the present findings cannot establish that mechanical energy was transferred from the hip to the ankle. Differences were detected only at maximum knee flexion and not at IC or peak vGRF, indicating clear event specificity rather than an effect arising during the earliest impact phase. The internal knee extension moment did not differ significantly, suggesting that no analogous condition-related difference occurred at the knee. Internal hip adduction, knee varus, and ankle inversion moments likewise showed no significant differences, indicating that the observed kinetic differences were confined to selected sagittal-plane variables. Their divergence from previous reports of frontal-plane abnormalities in CAI may reflect differences in comparison groups, landing direction, and task demands and should not be interpreted as evidence that frontal-plane control is universally preserved in CAI (12, 17, 23).

The third hypothesis was partially supported for ankle-muscle activity. LG RMS activation was lower, whereas peak TA activation was higher, indicating muscle-specific and metric-specific differences between conditions. RMS amplitude represents activation across the entire landing-descent phase, whereas peak activation represents the maximum activation amplitude; these measures therefore capture distinct features of neuromuscular activity (34). The combination of lower LG RMS activation and higher peak TA activation indicates that the two sEMG measures did not change uniformly across muscles, but it does not demonstrate a direct shift from sustained plantarflexor activity to transient dorsiflexor recruitment. Feger et al. (44) reported that lower-extremity muscle activation during functional tasks may be reduced in individuals with CAI, although this effect is not consistent across all muscles. Altered ankle-muscle activity during single-leg landing has likewise been reported in individuals with functional ankle instability (12, 45). Collectively, these findings support the view that neuromuscular differences in CAI may be specific to particular muscles and analytical windows rather than uniformly expressed across all ankle musculature. Lin et al. (17) reported lower PL activation during forward jump landing in athletes with CAI, whereas neither RMS nor peak PL activation differed between conditions in the present study. This inconsistency may arise from differences in comparison groups, horizontal momentum, preparatory demands, and analytical windows. Moreover, higher peak TA activation cannot be interpreted as the source of the larger internal ankle plantarflexion moment, because a net joint moment integrates the actions of all muscles crossing the joint and of passive structures, whereas sEMG reflects only the electrical activity of the recorded muscles (18, 33). The third hypothesis was also partially supported for muscle activity and co-contraction associated with the knee. Peak VL activation was higher, whereas activation of the RF, BF, and ST and most knee- and ankle-related CCIs did not differ significantly. The quadriceps contribute importantly to resisting knee flexion and downward body momentum during single-leg landing (18). However, the higher peak VL activation observed here was not accompanied by detectable differences in knee angle or the internal knee extension moment and should therefore be interpreted as an sEMG difference without a corresponding change in the measured knee-mechanical outcomes. The absence of a comparable RF response may relate to its biarticular function across the hip and knee, whereas the monoarticular VL has a more concentrated role in knee-extensor braking (46). The lack of differences in BF and ST activation indicates that a generalized increase in hamstring activation was not observed. Van Deun et al. (31) showed that neuromuscular alterations in CAI may extend from the ankle to proximal musculature; the present VL finding is compatible with the possibility that proximal muscles also display condition-related sEMG differences. The PL–MG CCI was lower, whereas the other seven CCIs did not differ significantly, indicating a difference specific to this muscle pair rather than a uniform alteration across all measured pairs. A lower CCI for a single pair cannot be equated with reduced overall joint stability, which also depends on other muscles, passive tissues, joint configuration, and external loading (3, 47). Given that ankle angles, frontal-plane moments, and TTS showed no detectable differences, the lower PL–MG CCI should be interpreted as a muscle-pair-specific difference in co-contraction rather than as evidence of global failure of ankle control.

In aggregate, the dual-task condition was characterized by a higher peak vGRF, smaller hip flexion angles at peak vGRF and maximum knee flexion, and differences in selected net internal joint moments, muscle-activation measures, and the PL–MG CCI. By contrast, TTS, the measured knee and ankle angles, frontal-plane joint angles, and most kinetic and sEMG outcomes showed no statistically significant differences. These findings should therefore be interpreted as outcome- and event-specific condition-related changes rather than as definitive evidence of load redistribution or a compensatory control mechanism. Such complex, multi-joint modulations under cognitive loading further support the notion that unilateral CAI induces central sensorimotor adaptations, demanding a higher reallocation of cognitive resources to maintain dynamic stability even during unilateral tasks (7, 8). This interpretation is consistent with the updated model of CAI, which emphasizes interactions among sensory–perceptual deficits, motor–behavioral deficits, and task and environmental constraints (3). From an applied perspective, the concurrent changes in peak vGRF, specific sagittal-plane hip postures, and localized muscle co-contraction (e.g., PL-MG) should not be viewed as isolated phenomena. Rather, they likely represent a coupled, non-linear neuromuscular control strategy where individuals with CAI attempt to modulate impact and maintain joint stability under working-memory constraints. However, standard univariate comparisons of discrete variables inherently limit our ability to fully decode these complex interrelationships. Recent methodological advancements highlight that combining multidimensional signals into integrated representations significantly improves the interpretation of functional instability. For instance, novel evaluation frameworks utilizing machine learning-based feature fusion have demonstrated superior capability in capturing the hidden correlations among kinematics, kinetics, and sEMG to identify CAI under various conditions (48). Furthermore, integrating external biomechanical data with deep learning to compute internal ligament loading mechanisms offers a powerful, data-driven approach to translating surface motor behaviors into underlying structural stresses (49). Future studies should transition from describing discrete surface differences to adopting these integrated, multimodal analytical frameworks to better decode the holistic sensorimotor and tissue-level adaptations in CAI. Clinically, however, inferences must be drawn with extreme caution. The observed coupled changes—particularly the altered sagittal hip posture and specific ankle co-contraction—represent task-specific adaptations to the immediate demands of working-memory load during high-impact landings, rather than definitive indicators of global functional failure. While existing evidence suggests that dual-task training may improve postural stability (11), the cross-sectional design of the present study precludes using these immediate biomechanical adjustments to directly dictate clinical rehabilitation protocols. Prospective monitoring is required to establish whether these task-specific movement strategies genuinely correlate with an increased risk of recurrent sprains.

This study has three principal limitations. First, no healthy control group was included; consequently, the findings characterize only within-participant responses to cognitive loading in individuals with CAI and cannot establish whether the observed pattern is specific to CAI. Second, joint angles and moments were extracted at only three discrete events. While this discrete approach is standard practice, it inherently fails to reflect the continuous nature of landing biomechanics. Continuous waveforms (e.g., using one-dimensional statistical parametric mapping), joint power, joint work, and joint stiffness were not analyzed, which limits our understanding of the continuous motor control strategies across the entire landing phase. The observed hip-ankle moment pattern therefore cannot be used to infer interjoint redistribution of mechanical energy. Third, the sample was relatively small and consisted exclusively of male university students with recreational-sport backgrounds; caution is therefore warranted when generalizing the findings to women, other age groups, or athletes with higher training status.

## 5 Conclusion

During single-leg drop landing in participants with CAI, the addition of a concurrent auditory working-memory task elicited specific, localized adaptations rather than a uniform failure of landing control. Specifically, the dual-task constraint was associated with a higher early impact, task-specific sagittal-plane compensations (smaller hip flexion angles), and targeted neuromuscular adjustments, such as reduced PL-MG co-contraction. These task-specific findings underscore that cognitive loading during high-impact movement modifies select biomechanical load-attenuation and muscular coordination strategies, highlighting the necessity of context-specific assessments in CAI research. Future studies should incorporate cognitive-task performance, larger and more diverse samples, and continuous biomechanical analyses to determine the clinical and prognostic significance of these findings.

## Data Availability

The raw data supporting the conclusions of this article will be made available by the authors, without undue reservation.
